# Indicators of In-Hospital Mortality in Infective Endocarditis: A Single-Center Retrospective Study

**DOI:** 10.7759/cureus.101308

**Published:** 2026-01-11

**Authors:** Dalila M Sacic

**Affiliations:** 1 Cardiology, University Clinical Center of Serbia, Belgrade, SRB

**Keywords:** heart failure, infective endocarditis, in-hospital mortality, septic shock, surgery

## Abstract

Infective endocarditis (IE) is associated with severe complications and high in-hospital mortality, ranging from 15% to 30%. Indicators of fatal outcome include the development of heart failure, septic shock, local uncontrolled infection or periannular complications, *Staphylococcus aureus *as a causative agent, negative blood cultures, and unoperated patients. This retrospective study determined clinical features and the indicators of in-hospital mortality in a cohort of 96 patients hospitalized in the cardiology clinic of the university hospital from January 1, 2018, to January 1, 2022, with definite infective endocarditis. Two-thirds of the patients were male, with a mean age of 53.5 years. Subjects with a fatal outcome (17.7%) were dominantly male (82%), and significantly older than the survivor group (61.6 vs. 51.7 years; p=0.014). There was a significantly higher presence of chronic kidney failure and malignancies in the fatal outcome subjects. Most significant complications of IE were heart failure (94.1% of subjects with a fatal outcome and 16.7% of survivors, p<0.001) and septic shock (82.4% vs. 1.3%; p<0.001). Surgery was performed in 11.8% of subjects with a fatal outcome and 57.7% of survivors (p=0.001). Cardiogenic shock and septic shock were the most common causes of death (35.3% and 23.5%, respectively). This retrospective study indicated the need for early recognition of high-risk IE patients and conservative and surgical treatment in the prevention of complications and fatal outcomes.

## Introduction

Infective endocarditis (IE) is an inflammatory process of the endocardium, usually affecting native or artificial heart valves or intracardiac devices [1,2]. Other cardiac structures may also be affected, including the interventricular septum, tendinous chordae, mural endocardium, and Valsalva sinuses [3].

The incidence of IE varies from seven to 15 cases per 100,000 persons per year [4,5]. A large number of recently published studies confirmed a significant increase in incidence due to an aging population, a rise in the number of intravenous drug users, the increasing number of patients with artificial valves and intracardiac devices, the use of long-term intravenous lines, as well as invasive procedures [6-8].

Making a diagnosis is a real challenge for doctors due to the fact that IE often has an atypical presentation. Significant progress was made possible by the application of transthoracic echocardiography (TTE) and transesophageal echocardiography (TEE), cardiac computed tomography (CCT), and positron emission tomography (PET) [8]. Nuclear medicine methods (single photon emission computed tomography {SPECT}/CT with radiolabeled leukocytes and fludeoxyglucose f-18 {18F-FDG} PET/CT) are useful in detecting IE of artificial valves and intracardiac devices [9].

During the 2000s, modified Duke's criteria for diagnostic classification based on the patient's clinical status, echocardiographic findings, and the results of the analysis of microbiological and pathohistological samples were recommended [10]. The mentioned variables are divided into the following two groups: large and small Duke's criteria. Patients who have fulfilled two major criteria, one major and three minor, or five minor criteria are treated as having a definite diagnosis of IE. The criteria were further modified in 2023, including new microbiology diagnostics, imaging (18F-FDG PET/CT), and inclusion of intraoperative inspection as a new major criterion [11].

Subacute IE (SBE) is most often caused by streptococci (especially viridans group, microaerophilic and anaerobic streptococci, and non-enterococcal group D streptococci and enterococci), and is less often caused by *Staphylococcus aureus, Staphylococcus epidermidis,* and anaerobic Haemophilus spp. SBE often occurs on altered valves, and after asymptomatic bacteremia due to periodontal, genitourinary, or gastrointestinal infection [12]. Prosthetic valve endocarditis (PVE) accounts for 10-30% of all patients with endocarditis. The incidence of PVE ranges from 0.3% to 1.2% [13]. Men older than 60 years with an artificial aortic valve are most often affected. The early form of PVE is acquired during the first year after surgery. In the early form of PVE, the infection can develop in the first two months after surgery, most often as a result of contamination during the procedure, with bacteria resistant to antibiotics, such as *Staphylococcus epidermidis*, diphtheroids, coliform bacilli, Candida spp.*, *andAspergillus spp.* *[14]. After that, PVE usually has the same pathogenesis, but it may also result from transient bacteremia unrelated to surgery [13]. IE, after transcatheter aortic valve implantation (TAVI) is more common in men, diabetic patients, and patients with moderate to severe aortic regurgitation. The diagnosis is difficult due to the atypical clinical picture and a large number of comorbidities. In-hospital mortality can also be influenced by the infection of the pacemaker electrode, which is often implanted in these patients [15]. Nosocomial (hospital-acquired IE) accounts for 5-29% of all causes of IE (excluding infections related to cardiac surgery). Infected intravascular catheters are the cause in 45-65% of patients, while urogenital and gastrointestinal diagnostic or surgical procedures are in second place. The most common cause of nosocomial IE is Gram-positive cocci; the course is usually acute, with a new or changed tumor in the area of the affected valve, and the mortality is high (from 40% to 56%) [16].

The prognosis of the IE is influenced by patient characteristics, type of causative agent (unfavorable prognosis in IE caused by *Staphylococcus aureus*, fungi, and non-Haemophilus, Aggregatibacter, Cardiobacterium, Eikenella, and Kingella {HACEK} Gram-negative bacilli), initial echocardiographic findings, and development of complications [17]. In recent studies, the long-term survival rate at the end of treatment has been estimated to be 80-90% in the first year, 70-80% in the second year, and 60-70% in the fifth year. Patients who cannot be operated on because of an unacceptably high risk have a poor prognosis [18]. Infective endocarditis is associated with severe complications and high in-hospital mortality [19]. Intrahospital mortality of IE ranges from 15% to 30% and increases with the number of predictors of negative outcome of IE [9,20].

Factors associated with an increased risk of in-hospital mortality in IE include the development of heart failure, septic shock, local uncontrolled infection or periannular complications (valve destruction or perforation, increased vegetation size, abscess, pseudoaneurysm, valve aneurysm or intracardiac fistula), causative agent *Staphylococcus aureus* or negative blood cultures, and unoperated patients [20,21]. Maintaining a positive blood culture result 48-72 h after starting antibiotic therapy indicates poor infection control and is an independent risk factor for in-hospital mortality [22]. Prosthetic endocarditis accounts for 10-30% of IE, with transcatheter-implanted valves having the highest mortality (47-64% for in-hospital and up to 75% for one-year mortality), primarily due to the older age and greater burden of comorbidities in patients undergoing this type of intervention [23,24].

The increase in mortality is particularly high in the first few years after discharge from the hospital, and is explained by late complications, such as heart failure, higher risk of recurrence, and greater vulnerability of patients [21]. A large number of authors who have dealt with this topic conclude that surgical intervention has a protective effect in relation to hospital mortality and long-term prognosis in patients with IE [25-27]. The aim of this retrospective study was to determine epidemiological, clinical, laboratory, etiological, and echocardiographic features of patients hospitalized with definite infective endocarditis, as well as to identify factors associated with in-hospital mortality.

## Materials and methods

Study design, population, and location

This study was conducted as a descriptive single-center retrospective cohort study. The study included the analysis of the medical records of patients hospitalized with IE in one department of the Cardiology Clinic of the University Clinical Center of Serbia in the period from January 1, 2018, to January 1, 2022. Data were collected from electronic medical records from the hospital database system based on International Classification of Diseases, 10th Revision (ICD-10) codes (I33.0; I33.9; I38, I39.8, B37.6) in the first or second diagnostic position recorded from hospital admission to the discharge list [28].

Inclusion criteria were adult hospitalized patients with a definitive diagnosis of IE, as confirmed during hospitalization according to the modified Duke's criteria (two major criteria or one major criterion plus three minor criteria or five minor criteria) [10]. The major criteria were positive blood culture for microorganisms typical of IE or other germs that can cause IE, and positive echocardiography, CT, or PET CT findings. The minor criteria were predisposition to IE, fever 38°C, vascular phenomena, immunological phenomena, and microbiological evidence [10].

Exclusion criteria included patients who did not have a definitive diagnosis of IE according to the modified Duke's criteria or younger than 18 years of age. The final sample was divided into the following two groups: subjects who died during hospitalization and subjects who were discharged from the hospital.

Patients were distributed by gender and age, whether they had predisposing factors, comorbidities on admittance, whether causative agent was isolated by blood culture, IE localization, presence and type of complications, were there any indications for replacement of the native valve, performed surgery. IE confirmation data analysis included clinical features of IE during admission to the hospital for treatment, the laboratory parameters of inflammation (C-reactive protein), and etiological agents found in blood cultures.

Echocardiographic data were obtained by transthoracic (TTE) and/or transesophageal (TEE) echocardiography and included the presence of vegetation, vegetation number, vegetation size, vegetation location, existence of perivalvular abscesses, pseudoaneurysms, and cuspid perforation. Data from other diagnostic imaging methods for IE and distant metastatic infectious foci (such as CT/MR/PET scan) were also analyzed. Heart failure was diagnosed according to the European Society of Cardiology (ESC) 2016 guidelines [29], and septic shock was defined according to the Sepsis-3 guidelines [30]. Indications for surgery were defined according to the 2015 ESC guidelines for diagnosis and treatment of infective endocarditis [31].

Descriptive and analytical statistical methods were used in the research. Depending on the type of variables and the normality of the distribution, the data description is presented as n (%), arithmetic mean±standard deviation, or median (min-max). Among the methods for testing statistical hypotheses, the author used t-test, Mann-Whitney U test, chi-square test, and Fisher's exact probability test. Statistical hypotheses were tested at a statistical significance level (alpha level) of 0.05. All data were processed using IBM SPSS Statistics 22 (Armonk, NY: IBM Corp.) software package.

## Results

The study sample comprised 96 adult hospitalized patients with a definitive diagnosis of IE according to the modified Duke criteria. In 17 (17.7%) subjects, the disease ended with a fatal outcome. When analyzing the whole sample, the majority of patients (n=64) were male (66.7%), with a male-to-female ratio of 2:1. The mean age was 53.5 years. Subjects with a fatal outcome were dominantly male (n=14; 82%), and significantly older than the survivor group (61.6 vs. 51.7 years; p=0.014).

There were no significant differences in the prevalence of smoking between these two groups, but in many subjects, in the fatal outcome (82.4%) and survivors (60.8%), the smoking status was not registered. The main predisposing factor in the whole sample and individual groups identified was congenital and/or acquired heart defect (n=87; 90.6%), followed by the presence of a prosthetic valve (n=13; 13.5%). Subjects with a fatal outcome and survivors most often had a native valve (82.4% vs. 81.8%, respectively). There is no statistically significant difference in the frequency of valve type in relation to outcome (Fisher's exact test, p=1.0).

In the survivors group, almost 26% (n=20) had some recent medical intervention (tooth extraction, appendectomy, dialysis catheter implantation), while 18% had confirmed intravenous drug addiction (n=14). Regarding comorbidities, 63% of the subjects had anemia (n=63), followed by arterial hypertension (n=42; 44%). There was a significantly higher presence of chronic kidney failure and malignancies in the fatal outcome subjects (Table 1).

**Table 1:** Distribution of subjects by demographics, predisposing conditions, and comorbidities. The chi-square test was used to calculate p-values for gender; the t-test was used for age; and Fisher's exact test was used for all other variables. IE: infective endocarditis; COPD: chronic obstructive pulmonary disease

Variables	Fatal outcome (n=17)	Survivors (n=79)	Total (n=96)	p-Value
Male, n (%)	14 (82.4)	50 (63.3)	64 (66.7)	0.130
Female, n (%)	3 (17.6)	29 (36.7)	32 (33.3)	
Age, mean±SD	61.6±13.2	51.7±17.8	53.5±17.4	0.014
Smoking				
smoker, n (%)	2 (66.7)	14 (45.2)	16 (47.1)	0.591
non-smoker, n (%)	1 (33.3)	17 (54.8)	18(52.9)	
Predisposing conditions				
Congenital and/or acquired heart defect, n (%)	15 (88.2)	72 (92.3)	87 (90.6)	0.631
Installed prosthetic valve, n (%)	2 (11.8)	11 (13.9)	13 (13.5)	1.000
Presence of intracardiac devices, n (%)	1 (5.9)	4 (5.1)	5 (5.2)	1.000
Medical interventions, n (%)	1 (5.9)	20 (25.6)	21 (21.9)	0.107
Intravenous drug addiction, n (%)	1 (5.9)	14 (17.9)	15 (15.6)	0.293
Previous IE, n (%)	1 (5.9)	7 (8.9)	8 (8.3)	1.000
Comorbidities				
Diabetes mellitus, n (%)	2 (11.8)	8 (10.3)	10 (10.4)	1.000
Anemia, n (%)	13 (76.5)	48 (61.5)	61 (63.5)	0.245
Arterial hypertension, n (%)	5 (29.4)	37 (46.8)	42 (43.8)	0.189
COPD, n (%)	3 (17.6)	7 (9.0)	10 (10.4)	0.378
Cerebrovascular insult, n (%)	2 (11.8)	6 (7.6)	8 (8.3)	0.628
Chronic kidney failure, n (%)	8 (47.1)	14 (17.9)	22 (22.9)	0.022
Malignancy, n (%)	3 (17.6)	1 (1.3)	4 (4.2)	0.017

Considering clinical features, dominant symptoms in both groups were general weakness and dyspnea, with the latter significantly more present in the fatal outcome group (76.5% vs. 27.8%) (Table 2). On admission, the most prominent signs included fever and the newly detected heart murmur. The altered mental state was significantly more common in the subjects who had fatal outcomes (47.1% vs. 13.9%; p= 0.005). The median value of CRP of subjects with a fatal outcome was 83.5 mg/L (range: 37.9-174.5 mg/L), while in surviving subjects it was 64.4 mg/L (range: 3.3-297.3 mg/L), with no significant difference in comparison to patients who had a favorable outcome (U=301.0; p=0.168).

**Table 2:** Distribution of symptoms and signs in relation to outcome. Chi-square test was used for calculating p-value for malaise and dyspnea, Fisher's exact test for all other variables.

Variables	Fatal outcome	Survivors	p-Value
Symptoms			
Exhaustion, n (%)	11 (64.7)	46 (58.2)	0.433
Dyspnea, n (%)	13 (76.5)	22 (27.8)	<0.001
Muscle/joint pain, n (%)	0 (0.0)	13 (16.5)	0.116
Chest pain, n (%)	4 (23.5)	10 (12.7)	0.265
Pain in the spine, n (%)	0 (0.0)	10 (12.7)	0.201
Dry cough, n (%)	2 (11.8)	15 (19.0)	0.729
Signs			
Fever, n (%)	17 (100.0)	71 (89.9)	0.343
Altered mental status, n (%)	8 (47.1)	11 (13.9)	0.005
Loss of body weight, n (%)	3 (17.6)	13 (16.5)	1.000
New heart murmur, n (%)	11 (64.7)	46 (58.2)	0.622
Janeway lesions, n (%)	1 (5.9)	1 (1.3)	0.324
CRP, median (mg/L)	83.5	64.4	0.168

In subjects with a fatal outcome, two major Duke criteria were present in 82.4% (n=14), while one major and three minor Duke criteria were present in 17.6% (n=3). Among the surviving subjects, 78.2% had two major Duke criteria (n=61), while 21.8% had one major and three minor Duke criteria (n=17). There was no statistically significant difference in the frequency of Duke criteria in relation to the outcome of IE (Fisher's exact test, p=1.0).

A total of 82.4% of subjects with a fatal outcome (n=14) and 82.1% of survivors (n=64) had a positive blood culture for the diagnosis of IE, which was not statistically significant (Fisher's exact test; p=1.0). Although Enterococcus spp*.* was found in 29.4% of subjects with fatal outcomes (n=5) vs. 13.9% in the survivor group (n=11), the difference has not reached statistical significance (Table 3).

**Table 3:** Distribution of the causative agents in relation to the outcome. Chi-square test was used for calculating p-value for Staphylococcus, Fisher's exact test for all other variables. IE: infective endocarditis

IE causative agent	Fatal outcome, n (%)	Survivors, n (%)	p-Value
Staphylococcus	7 (41.2)	44 (55.7)	0.276
Streptococcus	1 (5.9)	14 (17.7)	0.295
Enterococcus	5 (29.4)	11 (13.9)	0.151
Other	3 (17.6)	4 (5.1)	0.103

All subjects in both groups had a positive echocardiographic finding in the diagnosis of IE, most often in the form of vegetation (n=84; 88%) localized on aortic valve (70.6% in fatal outcome vs. 45.6% in survivors group), followed by mitral valve (23.5% vs. 45.6%) and tricuspid valve (23.5% vs. 20.3%) (Table 4).

**Table 4:** Distribution of IE localization and echocardiographic findings in relation to outcome. Chi-square test was used for calculating p-value for mitral and aortic valve, Fisher's exact test for all other variables. IE: infective endocarditis

Variables	Fatal outcome, n (%)	Survivors, n (%)	p-Value
Localization of IE			
Mitral valve	4 (23.5)	33 (41.8)	0.161
Aortic valve	12 (70.6)	36 (45.6)	0.061
Tricuspid valve	4 (23.5)	16 (20.3)	1.000
Pulmonary valve	0 (0.0)	2 (2.5)	1.000
Pacemaker	0 (0.0)	3 (3.8)	1.000
Echocardiographic finding			
Vegetation	15 (88.2)	69 (87.3)	1.000
Abscess	2 (11.8)	14 (17.7)	0.728
Perforation of the cusp	3 (17.6)	20 (25.3)	0.755
Pseudoaneurysm	0 (0.0)	5 (6.4)	0.581

No subjects with a fatal outcome had a positive PET-CT (vs. 9% of survivors, n=7), while 29.4% (n=5) had CT findings used as an IE diagnostic criterion (vs. 33.3% of survivors, n=26). Surgery was performed in 11.8% of subjects (n=2) with a fatal outcome and 57.7% of survivors (n=45), which is a statistically significant difference (chi-square test=11.778; p=0.001).

Most significant complications of IE were heart failure (94.1% of subjects with a fatal outcome, n=16 vs. 16.7% of survivors, n=13; chi-square test=39.480; p<0.001) and septic shock (82.4% of subjects with a fatal outcome, n=14 vs. 1.3% of survivors, n=1; Fisher's exact test, p<0.001).

Cardiogenic shock (n=6) and septic shock (n=4) were also the most common causes of death in the fatal outcome group (35.3% and 23.5%, respectively). Other causes included disseminated intravascular coagulation (n=3; 17.6%), thromboembolism (n=3; 17.6%), and acute renal failure (n=1; 5.9%).

## Discussion

This study explored several aspects of IE, such as symptoms, clinical signs, imaging, echocardiography, laboratory findings, and microbiological results. According to the results of our research, the characteristics of patients treated for IE in our cardiology unit match the characteristics of patients treated for IE in health institutions of the same level in European countries [32].

Epidemiological characteristics of IE have changed significantly along with the extension of life expectancy, the increase in the number of degenerative valvular damage, the significant increase in the number of patients with implanted mechanical or biological valves, as well as the increasingly frequent implementation of intracardiac devices and transcatheter aortic valve implantation (TAVI), and due to the increase in incidence nosocomial infections [5,15,33].

The data obtained from our research indicate that the patients who had a fatal outcome were significantly older. The data obtained are in agreement with studies published so far, which suggest that while the age limit for IE can be different (ranging from 36 to 69 years), the IE incidence increases significantly with increasing age [25]. On the other hand, multiple large-scale studies and systematic reviews confirm that advanced age significantly increases the risk of in-hospital mortality in patients with infective endocarditis (IE), independent of other factors such as comorbidities or surgical intervention [5,34-36].

Shah et al. indicate that the risk of the disease increases five times after the age of 80 years, which is associated with the frequency of degenerative changes in the valvular apparatus [25], while Khan et al. as well as Ursi et al. observe that in people over 70 years of age, there is a significant frequency of bacteria from the gastrointestinal tract as a cause of IE, such as Streptococcus group D*, S. bovis *(*Streptococcus gallolyticus*)*, Enterococcus faecalis*, etc. [37,38]. The same authors suggest that the risk of death in this age group continues to increase significantly with age, up to 28%. In accordance with the results of previously published research, in our study, IE patients were twice as often men as women [39].

Although IE can also occur in people who have not previously been diagnosed with heart disease, the existence of a heart defect is one of the most significant predisposing factors for the occurrence of IE. In our research, 88.2% (n=15) of subjects with a fatal outcome had a congenital and/or acquired heart defect. The presence of previous heart disease had no effect on the outcome.

Dominant signs in subjects that had a fatal outcome were fever, dyspnea, and altered mental state. Statistical data processing revealed an even distribution of other symptoms, such as pain in the muscles, chest, spine, and dry cough. The results of this study are consistent with the results of similar earlier clinical-epidemiological studies that included patients with IE [32,40]. The obtained results of this study indicate that the most common localization of IE was on the native valves (n=77; 81.9%), of which the aortic valve was affected by infective endocarditis in 70.6% of patients with a fatal outcome (n=12).

Establishing the diagnosis of IE requires the integration of clinical, laboratory, and echocardiographic data. The clinical diagnosis of IE based on Duke's criteria was established in all patients who participated in our research, and on the basis of two major criteria, it was established in more than three-quarters of the patients (n=75; 78.9%), while in 21.1% of patients (n=20) it was established based on one major and three minor criteria.

In accordance with the recommendations of the European Society of Cardiology (ESC) for the treatment of patients with IE, echocardiography is still the gold standard in the diagnosis of IE, and in our study, it was performed in all patients. With this method, in our research, vegetations were recorded in 88.2% of subjects with a fatal outcome (n=15), which is in accordance with the data on the sensitivity of this method presented in the available literature [24,41]. Since the risk of embolism in patients with IE is related to the size, morphology, and mobility of the vegetation, proper assessment of the vegetation and monitoring of its size from the outset and during antibiotic therapy have important prognostic implications [42-44]. Other investigated echocardiographic characteristics of the subjects in this study, such as the occurrence of abscess, cuspid perforation, and pseudoaneurysm, had no influence on the outcome.

Blood cultures are the most important laboratory diagnostic test in IE. In our study, 82.4% (n=14) had a positive blood culture result with a fatal outcome. A negative finding was recorded in 17.6%, which can be attributed to the early initiation of antibiotic therapy and the specific causative agent. Similar results can also be found in other published studies, which are related to the use of antibiotics during the first two weeks after the onset of the first febrile illness [45]. Cases of endocarditis with negative blood cultures fall into one of the following categories: sterile blood cultures due to previous antibiotic use; slow growth of certain microorganisms, such as HACEK bacteria, certain species of Candida, and endocarditis caused by intracellular organisms, such as Bartonella (various species), Chlamydia, and *Tropheryma whipplei* [46]. In a prospective study of blood culture-negative endocarditis, the causative microorganism was serologically identified in almost half of the cases, with the presence of *Coxiella burnetii* and Bartonella identified as the most common pathogens [47]. Non-bacterial thrombotic (marantic) endocarditis can be a consequence of autoimmune or neoplastic diseases. One must also think about the self-initiated taking of antibiotics, which, as a practice in our environment, is not a rare occurrence. In the literature, some authors attribute the negative blood culture result to the difficulty of isolating certain microorganisms, such as HACEK group microorganisms,Legionella spp., Mycoplasma spp.*,* or intracellular pathogens [48]. Accordingly, some authors advise that, in addition to the recommended analyses, the routine examination should also include an analysis of the presence of antibodies against microorganisms, such as *Coxiella burnetii* and Bartonella, followed by polymerase chain reaction (broad range, valvular biopsies) [49].

The complexity of diagnostic and therapeutic procedures is linked to the increase in the frequency of IE caused by *Staphylococcus aureus*, which is found in 30% of patients [33]. IE caused by this Staphylococcus has a more aggressive form, with persistent bacteremia and a higher risk of embolic complications. *Staphylococcus aureus* is also the most common cause of IE in artificial valves, when, as a rule, reintervention is required, and mortality ranges up to 50%. The frequency of IE caused by coagulase-negative Staphylococcus increased by 10% [50]. It is a frequent cause of IE of artificial valves in the first year after the intervention. This Staphylococcus is often methicillin-resistant [51].

In our study, Staphylococcus was the cause of IE in 41.2% of subjects with a fatal outcome (n=7), and Enterococcus was the cause of IE in 29.4% of patients with a fatal outcome (n=5), but without a statistically significant difference in the effect of the causative agent on the outcome. The frequency of Enterococcus spp. as a cause of IE is associated with increased exposure to invasive procedures, especially of the urinary and gastrointestinal tracts [37].

Of the comorbidities, the presence of chronic kidney failure and malignancy influenced the outcome. A total of 47.1% of subjects with a fatal outcome had chronic kidney failure as a comorbidity. Malignancy as a comorbidity was present in 17.6% of subjects with a fatal outcome (n=3). Along with the aforementioned data that mortality is higher in the elderly, this research confirms that mortality is higher in the elderly with comorbidities [4-6]. Complications in patients with IE can result from infection or embolic manifestations, but they can also arise from adverse effects of the administered drug or from the host's immune response to the infection. Of the total number of patients with a fatal outcome in our study, the most common complication was heart failure (94.1%), and 82.4% of subjects had septic shock as a complication. The results obtained in this way confirmed that the development of heart failure and septic shock has a significant impact on in-hospital mortality [20,21,52].

If the control of the disease has not been established by antimicrobial treatment, and as a result, acute complications arise, or there is a high risk for the development of complications of the disease, urgent surgical treatment is considered, by means of which the vegetation is removed, and the natural valve is replaced with an artificial one [53]. In our research, 11.8% of subjects with a fatal outcome (n=2) and 57.7% of survivors (n=45) had surgical intervention. Surviving respondents had surgery significantly more often, which confirms this hypothesis. Surgical procedures differ according to the degree of urgency and, in the case of IE, can be elective (planned), which are performed in patients who have been previously treated with antimicrobial drugs according to the recommended protocol for the full duration and doses, or can be urgent. Emergency surgical interventions in the case of IE must be performed within seven days of setting the indication [54]. Surgical interventions of the highest degree of urgency are performed within 24 h of the indicated indication in patients with acute cardiac arrest or new severe regurgitation. It is recommended that blood cultures be therapeutically negative before surgery in order to reduce the risk of septic complications. If not treated adequately, IE is a disease that ends in death. Timely and adequate antibiotic therapy reduces the mortality rate by 20-50%, while cardiac surgical treatment of patients reduces mortality by an additional 20-50% [5,33].

The mortality of patients in our study was 17.7%, which is actually the middle limit of the mortality range recorded in developed countries of the Western world (15-22%), and significantly lower than the mortality of patients with IE recorded in less developed parts of the world, such as Latin American countries and Asia where mortality is recorded in the range of 31-46% of IE sufferers [35].

Limitations

The main limitation of the study is the relatively small number of subjects with the outcome of interest (fatal outcome), so due to numerical limitations, we were not able to perform multivariate analyses and/or detect predictors that others obtained in their research. Another limitation is the retrospective nature of the study and single department recruitment. Some data (e.g., smoking status) were not available for all included subjects. There is a possibility of survival bias concerning surgical intervention.

## Conclusions

This retrospective study indicated the need for early recognition of high-risk IE patients and conservative and surgical treatment in the prevention of complications and fatal outcomes. The timely recognition of age, gender, and comorbidities (chronic kidney failure, malignancies) as indicators of fatal outcome in the IE patients, as well as the most common causes of infection (Staphylococcus and Enterococcus spp.), could decrease the speed of development of heart failure and septic shock as the main causes of in-hospital mortality. Surgical treatment in indicated cases appears to have a positive effect on the favorable outcome of the disease.
